# MTA1 promotes epithelial to mesenchymal transition and metastasis in non-small-cell lung cancer

**DOI:** 10.18632/oncotarget.16404

**Published:** 2017-03-21

**Authors:** Ke Ma, Yangwei Fan, Xuyuan Dong, Danfeng Dong, Yuyan Guo, Xin Wei, Jing Ning, Qianqian Geng, Chuying Wang, Yuan Hu, Mengya Li, Wenxia Niu, Enxiao Li, Yinying Wu

**Affiliations:** ^1^ Department of Medical Oncology, The First Affiliated Hospital of Xi'an Jiaotong University, Xi'an, Shaanxi 710061, P.R. China; ^2^ Department of Medical Radiation Oncology, The First Affiliated Hospital of Xi'an Jiaotong University, Xi'an, Shaanxi 710061, P.R. China; ^3^ Department of Medical Oncology, Shaanxi Province People's Hospital, Xi'an, Shaanxi 710068, P.R. China; ^4^ Department of Obstetrics and Gynecology, Xi'an Third Hospital, Xi'an, Shaanxi 710068, P.R. China; ^5^ Department of Nuclear Medicine, The First Affiliated Hospital of Xi'an Jiaotong University, Xi'an, Shaanxi 710061, P.R. China

**Keywords:** MTA1, metastasis, epithelial to mesenchymal transition, NSCLC, AKT

## Abstract

The present study assessed the role of metastasis-associated protein 1 (MTA1) in epithelial to mesenchymal transition (EMT) and metastasis in non-small-cell lung cancer (NSCLC) cells using a normal lung epithelium cell line, three NSCLC cell lines, a mouse NSCLC model, and 56 clinical NSCLC samples. We observed that MTA1 overexpression decreased cellular adhesion, promoted migration and invasion, and changed cytoskeletal polarity. MTA1 knockdown had the opposite effects. MTA1 overexpression decreased E-cadherin, Claudin-1, and ZO-1 levels and increased Vimentin expression *in vitro* and *in vivo*, through activation of AKT/GSK3β/β-catenin signaling. However, treatment with the AKT inhibitor MK2206 did not completely rescue effects associated with MTA1 expression changes, indicating that pathways other than the AKT/GSK3β/β-catenin pathway could be involved in MTA1-induced EMT. Compared with normal lung tissues, MTA1 expression was elevated in NSCLC patient tissues and was correlated with American Joint Committee on Cancer stage, T stage, lymphatic metastasis, and patient overall survival. Additionally, MTA1 expression was positively associated with p-AKT and cytoplasmic β-catenin levels. These findings indicate MTA1 promotes NSCLC cell EMT and metastasis via AKT/GSK3β/β-catenin signaling, which suggests MTA1 may be an effective anti-NSCLC therapeutic target.

## INTRODUCTION

Lung cancer is one of the most common malignant cancers and is the leading cause of cancer-related mortality worldwide [1]. Epithelial to mesenchymal transition (EMT) is a key step in the invasion and metastasis processes in human cancers [2–4]. The molecular mechanisms responsible for non-small cell lung cancer (NSCLC) metastasis are not fully understood. During EMT, epithelial cells lose their epithelial characteristics and assume invasive and migratory mesenchymal phenotypes, enabling them to leave the tissue parenchyma and enter the systemic circulation [4, 5]. EMT is commonly observed in lung cancer cells and may be a potential target for lung cancer therapy [6]. Several inhibitors targeting critical orchestrators at the convergence of EMT pathways are under preclinical and clinical investigation [7].

Metastasis-associated gene 1 (MTA1), an essential component of the nucleosome remodeling and deacetylase (NuRD) complex, appears to promote cancer progression and metastasis [8–12]. MTA1 is overexpressed in a variety of human cancers, including NSCLC [13–16], providing a new molecular target for anti-cancer therapeutics. MTA1 reportedly contributes to tumor metastasis by promoting EMT in some cancers [17–21]. However, no study has systematically investigated MTA1 functions in the NSCLC EMT and metastasis processes.

The present study evaluated the biological functions and clinical significance of MTA1 using a normal lung cell line, three NSCLC cell lines, tissues from a mouse NSCLC model, and clinical NSCLC samples. We demonstrated that MTA1 promotes NSCLC cell metastasis *in vitro* and *in vivo* by encouraging the EMT and activating AKT/GSK3β/β-catenin signaling.

## RESULTS

### MTA1 is differentially expressed in NSCLC cell lines

**Figure 1:**
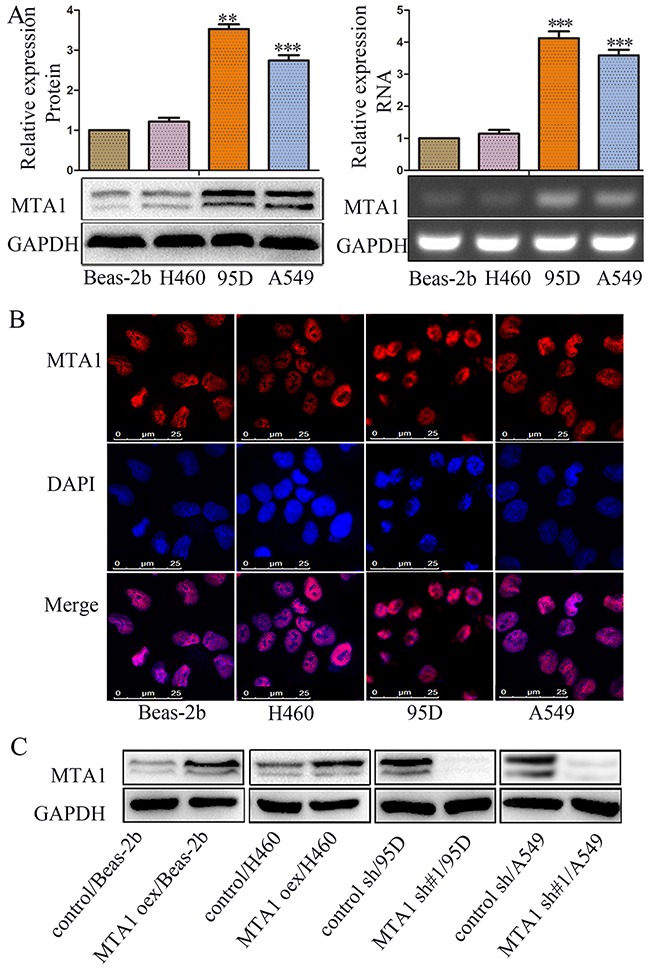
MTA1 expression and cellular location in NSCLC cell lines **(A)** Western blot(left) and RT-PCR (right) analyses of MTA1 expression in four cell lines. **P<0.01, ***P<0.001, compared with Beas-2b cells. GAPDH was used as loading control. Student t-test was used for statistical analyze for three independent experiments. For PCR amplification, 25 cycles were performed. **(B)** Immunofluorescence staining for MTA1 in four cell lines (red: MTA1; blue: nuclei). **(C)** Western blot analysis of MTA1 expression in MTA1-overexpressing cells, MTA1-silenced cells, and control cells. GAPDH was used as a loading control. oex: overexpression; sh#1: shRNA#1.

With examined MTA1 expression in different NSCLC cells using RT-PCR and western blotting. MTA1 was differentially expressed in the four cell lines as follows: Beas-2b<H460<A549<95D (Figure 1A). MTA1 mRNA and protein levels were higher in 95D and A549 cells compared to Beas-2b and H460 cells (*P*<0.001). Variations in MTA1 localization have been observed in various cells and tissues [22]. Immunofluorescence staining detected MTA1 in the cell nucleus (Figure 1B). We successfully upregulated MTA1 expression in Beas-2b and H460 cells via plasmid transfection, and downregulated MTA1 in 95D and A549 cells using lentivirus-mediated shRNA (Figure 1C).

### MTA1 upregulation promotes NSCLC cell invasion and migration *in vitro*

**Figure 2:**
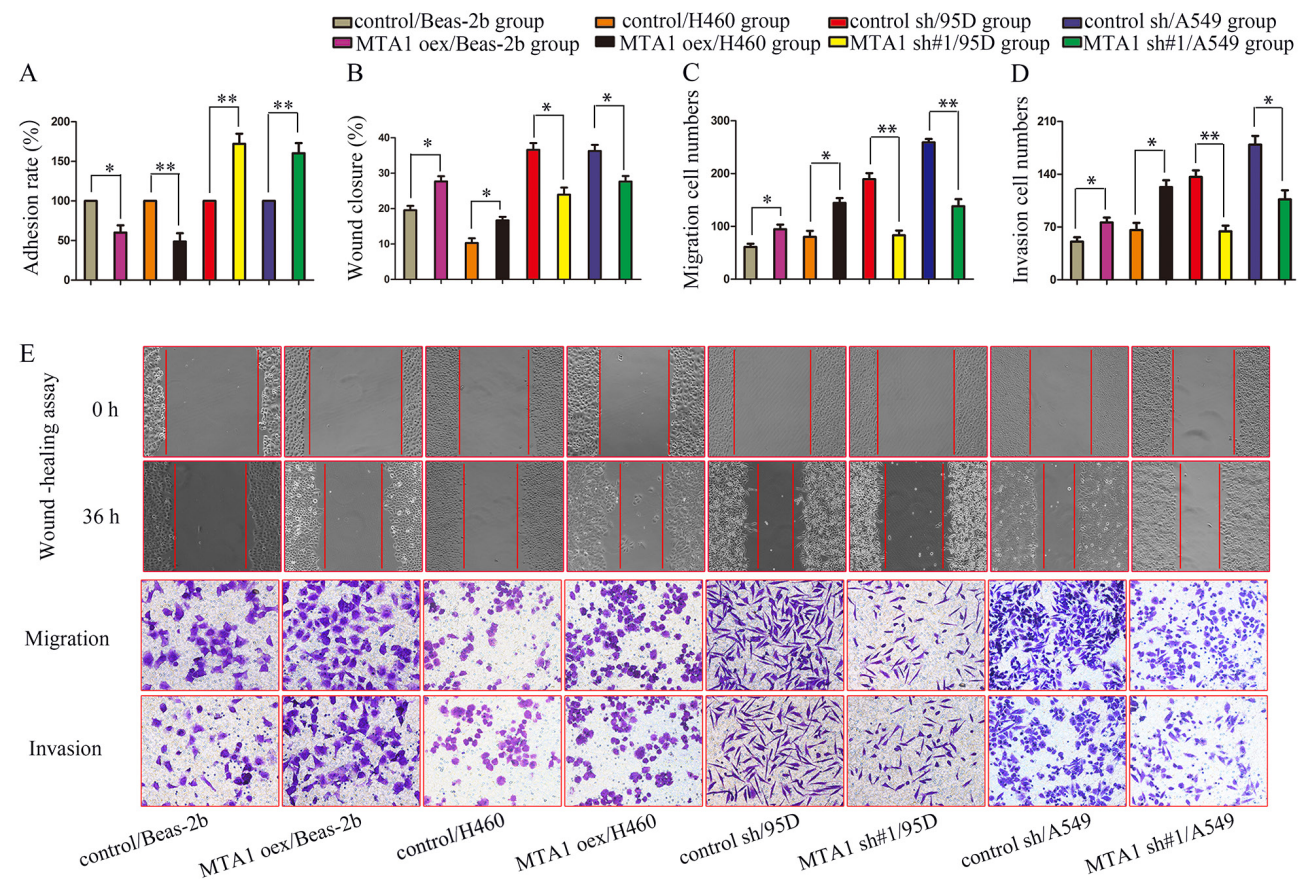
MTA1 expression is associated with NSCLC cell metastasis **(A)** Cell adhesion rate as measured using the MTT assay. **(B–E)** Quantified data **(B–D)** and representative images **(E)** of wound healing (upper), migration (middle), and invasion assays (bottom). oex: overexpression; sh#1: shRNA#1. Student t-test was used for statistical analyze for three independent experiments. *P<0.05, **P<0.01.

*In vitro* adhesion was increased in 95D and A549 cells treated with MTA1 shRNA compared to the same cell lines treated with control shRNA (Figure 2A). Similarly, adhesion was reduced in Beas-2b and H460 cells treated with the MTA1 overexpression plasmid compared to the same cells treated with empty plasmid (Figure 2A). We analyzed cell migration and invasion using wound-healing and transwell assays. 36 h after wound-healing assay scratches were made, cell-free areas in the MTA1 overexpression groups were smaller than those in the control groups (Figure 2B). Similarly, cell-free areas in the MTA1 shRNA groups were larger than those in the control groups (Figure 2E). Transwell assays showed that MTA1 upregulation promoted cell migration (Figure 2C & 2E) and invasion (Figure 2D & 2E) *in vitro*, while MTA1 downregulation inhibited cell migration (Figure 2C & 2E) and invasion (Figure 2D & 2E). Together, these data indicated that MTA1 upregulation promotes NSCLC cell adhesion, migration, and invasion.

### MTA1 promotes EMT in NSCLC cells *in vitro*

**Figure 3:**
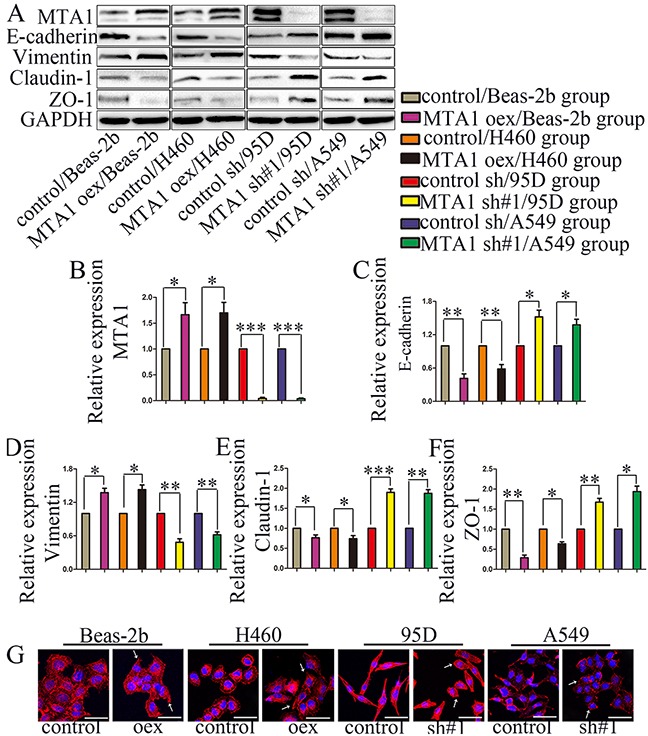
MTA1 expression is associated with NSCLC cell EMT **(A)** Western blot analysis of MTA1, E-cadherin, Vimentin, Claudin-1, and ZO-1 expression GAPDH was used as a loading control. Quantitative data are shown in **(B–F)**. Student t-test was used for statistical analyze for three independent experiments. *P<0.05, **P<0.01, ***P<0.001. **(G)** Cell cytoskeleton images were obtained via confocal microscopy MTA1 overexpression changed cells from relatively circular to irregular in shape, with the emergence of prolonged “feet.” The “feet” of MTA1-shRNA-treated cells were shortened, and cells changed from long spindles to relatively elliptical or circular in shape compared with controls. Red: F-actin; blue: nuclei (bars: 50 μm). →: representative changes; oex: overexpression; sh#1: shRNA#1.

**Figure 4:**
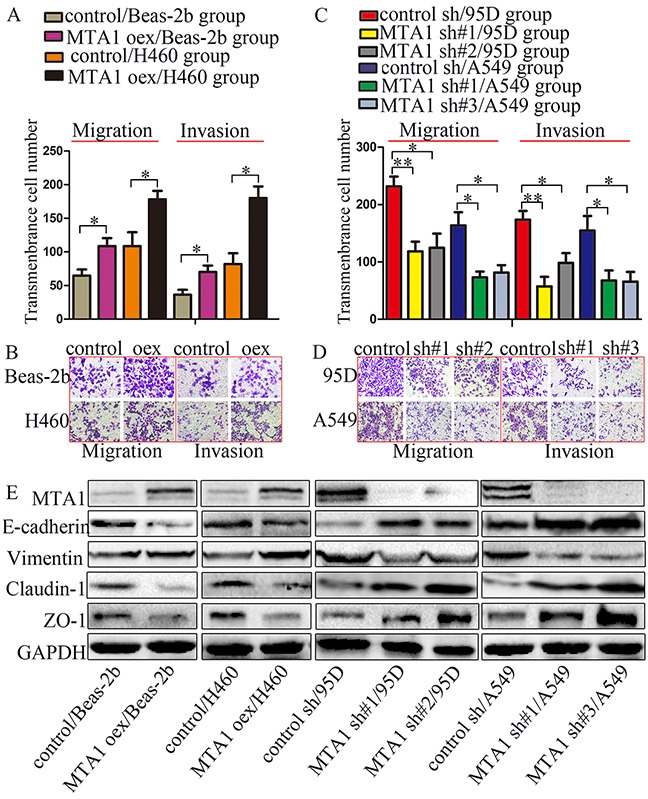
MTA1 promotes NSCLC cell metastasis through EMT Cells were transfected with MTA1-overexpression plasmid or infected with lentivirus containing shRNA for 72 h, and then were collected to perform the following experiments. Quantified data **(A)** and representative images **(B)** of migration (left) and invasion assays (right) in Beas-2b and H460 cells. Quantified data **(C)** and representative images **(D)** of migration (left) and invasion assays (right) in Beas-2b and H460 cells. Student t-test was used for statistical analyze for three independent experiments. *P<0.05, **P<0.01. **(E)** Western blot analysis of MTA1, E-cadherin, Vimentin, Claudin-1, and ZO-1 expression GAPDH was used as a loading control. oex: overexpression, sh#1: shRNA#1, sh#2: shRNA#2, sh#3: shRNA#3.

MTA1 reportedly promotes EMT [17–21]. We assessed whether MTA1-induced NSCLC cell migration and invasion was related to EMT. MTA1 overexpression decreased E-cadherin, Claudin-1, and ZO-1 levels, and increased Vimentin levels, and MTA1 knockdown reversed these effects (Figure 3A–3F). Cytoskeleton reorganization and polarity changes reportedly promote metastasis [23]. Therefore, we examined the effects of MTA1 overexpression and silencing on cytoskeleton structures using TRITC Phalloidin staining. MTA1 shRNA-treated cell “feet” were remarkably shortened and cells changed from long and spindle-shaped to relatively elliptical or circular compared with controls (arrow) (Figure 3G). Following MTA1 upregulation, cells changed from relatively circular to irregularly-shaped with the emergence of prolonged “feet” (arrow) (Figure 3G). In transiently infected/transfected cells, MTA1 upregulation increased cell metastasis, decreased expression of E-cadherin, Claudin-1, and ZO-1, and increased Vimentin levels (Figure 4A–4B & 4E). These effects were reversed following MTA1 silencing (Figure 4C–4D & 4E). The above results demonstrated that MTA1 promoted NSCLC cell metastasis by encouraging the EMT.

### MTA1 knockdown inhibits NSCLC metastasis by regulating EMT *in vivo*

**Figure 5:**
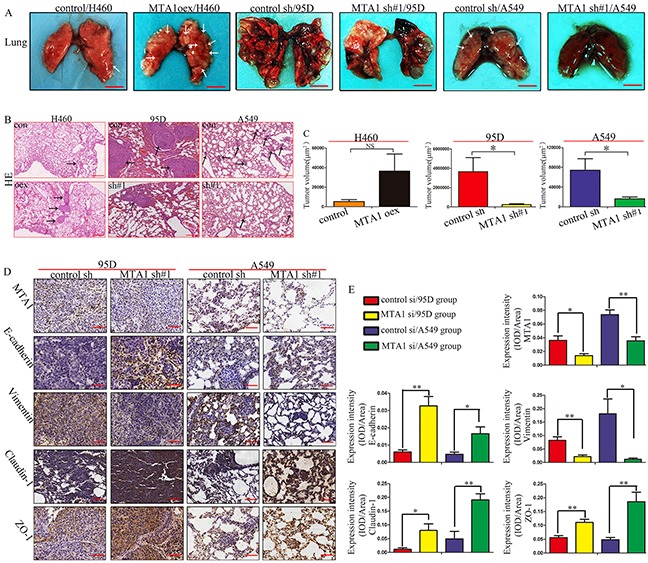
MTA1 knockdown inhibits lung metastasis by regulating EMT *in vivo* **(A)** Lung metastasis in mice after intravenous tail vail injection of cancer cells Bars: 5.0 mm. →: Suspicious metastasis lesions. **(B)** HE staining of lung sections Bars: 250μm. →: Confirmed metastasis lesions. **(C)** Quantitative analysis(n=5) of lung metastasis tumor volume, calculated when bars=250μm. **(D)** Immunohistochemical staining for MTA1, E-cadherin, Vimentin, Claudin-1, and ZO-1 in lung tissues. Bars=50μm. **(E)** Expression intensity (MTA1, E-cadherin, Vimentin, Claudin-1, ZO-1) was calculated as IOD/Area (n=5). oex: overexpression; sh#1: shRNA#1. Student t-test was used for statistical analyze for three independent experiments. NS: no significance, *P*>0.05. *P<0.05, **P<0.01, ***P<0.001.

We constructed a mouse xenograft model to further assess whether MTA1-induced NSCLC metastasis was associated with EMT. Mice injected with MTA1-overexpression plasmid-treated cells via the tail vein exhibited larger macroscopic metastases compared with controls, although this difference was not statistically significant (P>0.05) (Figure 5A–5B). In the untreated 95D cell group, mouse lung tissue lost much of its original structure, and contained large metastases. In the MTA1-shRNA-treated 95D cell group, lung tissue remained mostly clear, and tumors were smaller compared to controls (Figure 5B–5C). Untreated A549 cells mainly formed a lot of small metastases, and relatively more and larger than those in the MTA1-shRNA-treated A549 cell group (Figure 5B–5C). There were no obvious metastatic lung lesions in the MTA1-shRNA-treated A549 cell group, and only very small metastases were visible via HE staining (Figure 5A). Immunohistochemical staining (Figure 5D–5E) showed increased E-cadherin, Claudin-1, and ZO-1 levels, and decreased Vimentin levels in MTA1-shRNA-treated lung tissues.

### MTA1 promotes NSCLC cell EMT through AKT/GSK3β/β-catenin signaling

**Figure 6:**
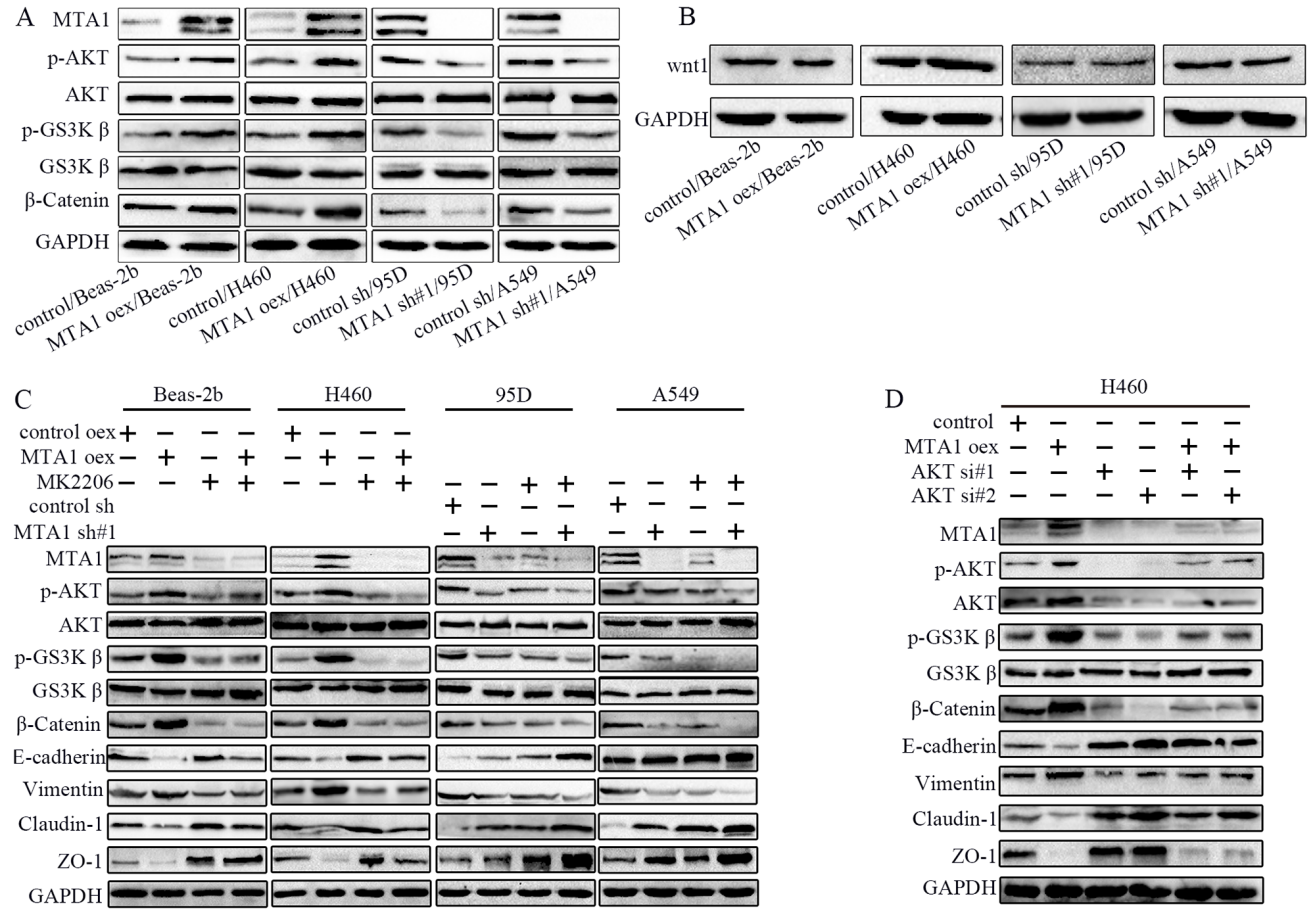
MTA1 promotes NSCLC cell EMT through the AKT/GSK3β/β-catenin signaling pathway **(A)** Western blot analysis of AKT, p-AKT, GSK-3β, p-GSK-3β, and β-catenin expression, **(B)** Western blot analysis of Wnt1. **(C)** Western blot analysis of MTA1, AKT, p-AKT, GSK-3β, p-GSK-3β, β-catenin, and EMT markers (E-cadherin, Vimentin, Claudin-1, and ZO-1). Cells were treated with 10 μM MK2206 for 24 h. **(D)** Western blot analysis of MTA1, AKT, p-AKT, GSK-3β, p-GSK-3β, β-catenin, and EMT markers (E-cadherin, Vimentin, Claudin-1, and ZO-1). H460 cells were transfected with MTA1-overexpression plasmid for 72 h, or transfected with siRNA for 48 h, or transfected with MTA1-overexpression plasmid for 24 h followed by siRNA for 48 h. GAPDH was used as a loading control. oex: overexpression; si#1: siRNA#1; si#2: siRNA#2.

The PI3K/AKT pathway is a central regulator of EMT [24, 25] and is constitutively activated in NSCLC cells [26]. MTA1 reportedly regulates E-cadherin expression by activating AKT, promoting prostate cancer cell invasion and metastasis [27]. MTA1 can also regulate GSK-3β expression [21], and β-catenin is a promising EMT-related therapeutic target [7, 28]. MTA1 upregulation increased, and downregulation decreased, p-AKT, p-GSK-3β, and β-catenin levels (Figure 6A). MTA1 was previously reported to activate Wnt signaling by regulating Wnt1 expression [29, 30]. GSK-3β acts at the convergence of the PI3K/AKT [31, 32] and Wnt pathways [33], both of which are critical in EMT [7, 34]. However, we found that neither MTA1 upregulation nor downregulation changed Wnt1 expression in NSCLC cells (Figure 6B). The results demonstrated that MTA1 promoted EMT by activating AKT/GSK3β/β-catenin signaling, but not Wnt1/GSK3β/β-catenin signaling.

To investigate the interaction between MTA1 and p-AKT, we treated cells with MK2206 and MTA1-overexpression plasmid or MTA1-shRNA. MTA1 knockdown-induced E-cadherin, Claudin-1, and ZO-1 overexpression was further enhanced by MK2206 treatment (Figure 6C). MTA1 overexpression decreased E-cadherin, Claudin-1, and ZO-1 levels, which were also blocked by MK2206 treatment in these cells (10μM, 24 h). Cumulatively, these results demonstrate that MTA1 promoted EMT by activating the AKT/GSK3β/β-catenin pathways. This was further verified by siRNA-mediated AKT knockdown in H460 cells (Figure 6D).

**Figure 7:**
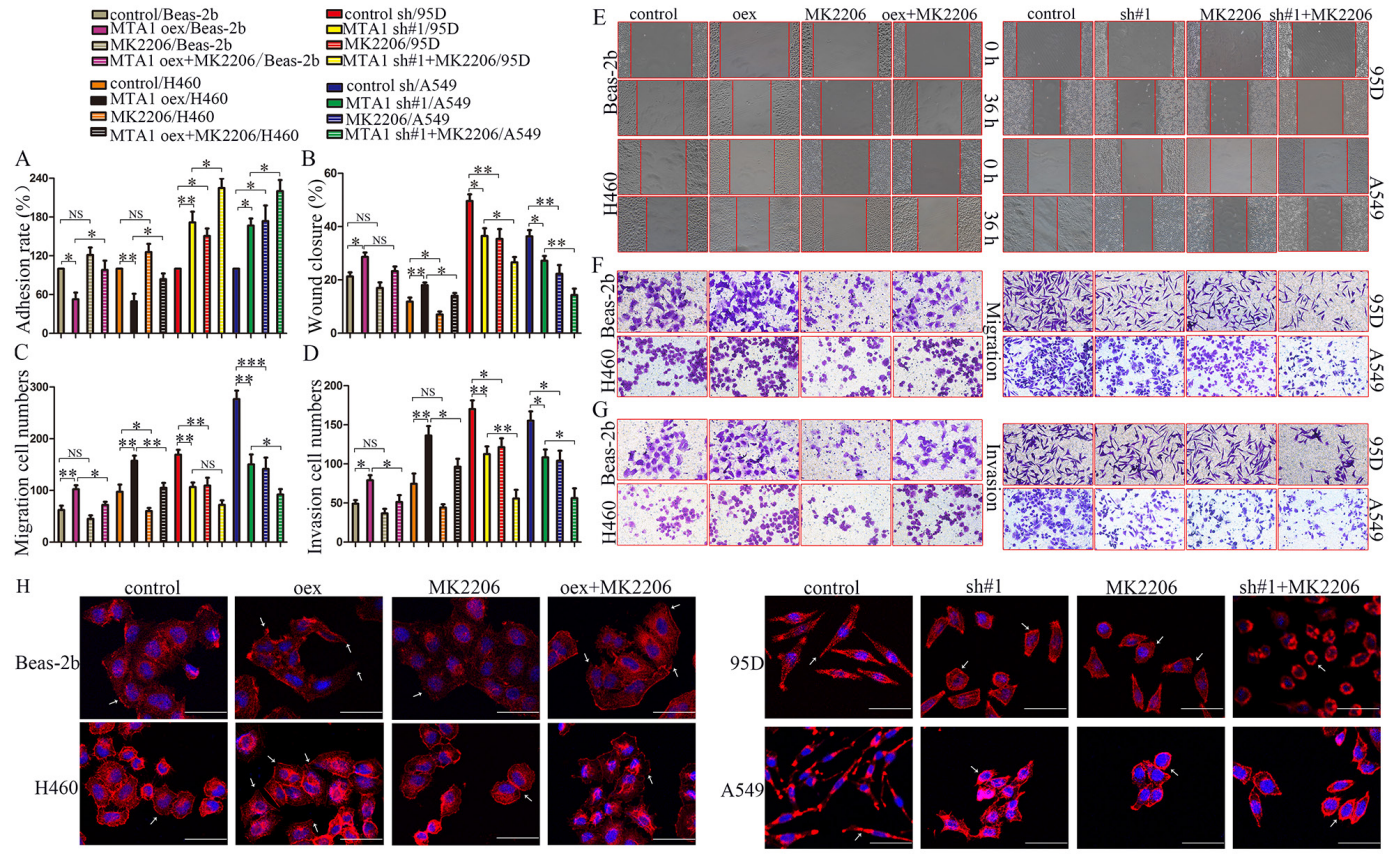
Effects of AKT inhibitor, MK2206, on MTA1-induced malignant phenotypes **(A)** Cell adhesion rate as measured using the MTT assay. Quantified data **(B–D)** and representative images of wound healing **(E)**, migration **(F)** and invasion assays **(G)** One-way ANOVA and LSD was used for statistical analyze for three independent experiments. NS: no significance, *P*>0.05. *P<0.05, **P<0.01, ***P<0.001. **(H)** Cell cytoskeleton images as analyzed using confocal microscopy Red: α-tubulin; blue: nuclei (bars: 50μm). oex: overexpression; sh#1: shRNA#1.

In the adhesion assay, MK2206 treatment reversed MTA1 overexpression-reduced adhesion and amplified MTA1-shRNA-increased adhesion (Figure 7A). The effects of MK2206 on MTA1-overexpression or -knockdown NSCLC cells were confirmed using wound healing and transwell assays (Figure 7B–7G). Wound healing was decreased in MTA1-overexpression Beas-2b normal lung epithelium cells treated with MK2206 compared with untreated cells, although this difference was not statistically significant (P>0.05, Figure 7B). We speculate that MTA1-AKT interaction mechanisms differ between normal and carcinoma cells. Unexpectedly, there was also no significant difference in 95D cell migration between the MTA1-shRNA and MTA1-shRNA + MK2206 groups (Figure 7C, P>0.05), although the number of transmembrane cells was reduced following MK2206 treatment. We thought this may due to the effect of MTA1 in 95D. MK2206 also induced morphological changes in treated 95D and A549 cells. MK2206 treated cell “feet” were remarkably shortened and cells changed from long and spindle-shaped to relatively elliptical or circular compared with controls (arrow) (Figure 7H). However, MK2206 induced morphological changes was not obvious in Beas-2b and H460 cells.

### MTA1 expression in NSCLC tissues is associated with patient clinicopathological characteristics

**Figure 8:**
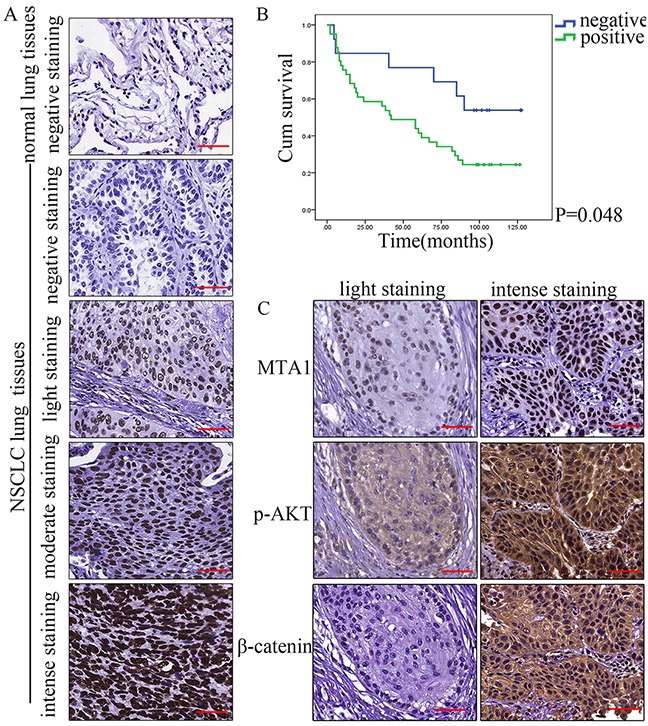
Clinical significance of MTA1 expression in NSCLC tissues **(A)** Representative immunohistochemical staining for MTA1 in NSCLC and normal lung tissues Top to bottom: normal lung; negative immunostaining in NSCLC tissues; light in NSCLC tissues; moderate in NSCLC tissues; intense in NSCLC tissues. Bars: 50μm. **(B)** Kaplan-Meier survival curves for MTA1 expression in 54 NSCLC patients Relationships between MTA1 expression and p-AKT and β-catenin expression in NSCLC tissues. **(C)** Representative immunohistochemical staining for MTA1, p-AKT, and β-catenin. Bars: 50μm.

Immunohistochemical staining showed MTA1 in nuclei in both NSCLC patient tissues and normal lung (Figure 8A). MTA1 expression was negative or weak in normal lung tissues, but was high in 73.2% of NSCLC patients. Associations between MTA1 expression and various patient clinicopathological characteristics are shown in Table 1. MTA1 expression was associated with American Joint Committee on Cancer (AJCC) TNM stage, T stage, and lymphatic metastasis.

**Table 1:** Associations between MTA1 expression and NSCLC patient clinicopathological characteristics

Variables	No.	Weak expression		High expression		χ2	P-value
		No.	%	No.	%		
Gender						0.286	0.713
Male	46	13	28.26	33	71.74		
Female	10	2	20.00	8	80.00		
Ages						3.810	0.051
≤60 years	27	4	14.81	23	85.19		
>60 years	29	11	37.93	18	62.07		
AJCC stage						7.496	***0.005***
I/II	41	15	36.59	26	63.41		
III/IV	15	0	0.00	15	100.00		
T stage						4.454	***0.048***
T1-2	46	15	32.61	31	67.39		
T3-4	10	0	0.00	10	100.00		
Lymphatic metastasis						4.462	***0.035***
Yes	28	4	14.29	24	85.71		
No	28	11	39.29	17	60.71		
Tumor type						0.759	0.684
SC	33	8	24.24	25	75.76		
AC	22	7	31.82	15	68.18		
ASC	1	0	0.00	1	100.00		

AJCC: American Joint Committee on Cancer.

SC: squamous carcinoma; AC adenocarcinoma; ASC: adeno-squamous carcinoma.

Significant *P*-values are bolded (*P*<0.05).

### MTA1 expression is associated with NSCLC patient survival

We assessed whether MTA1 expression could predict patient outcome. Two patients were excluded from the analysis due to death unrelated to the tumor. Univariate analyses revealed that AJCC stage, lymphatic metastasis, and MTA1 expression were associated with NSCLC patient overall survival (Table 2). Kaplan Meier survival curves for MTA1-positive and -negative cases are shown in Figure 8B. Multivariate analysis using a Cox proportional hazards model revealed that only lymphatic metastasis remained independently associated with overall survival (Table 3).

**Table 2:** Univariate analysis of NSCLC patient prognosis (n=54)

Variable	Survival rate (%)			mOS (months)	P-value
	3 years	5 years	8 years		
Gender					0.179
Female	70.0	70.0	50.0	84.0	
Male	63.6	47.7	27.3	58.0	
Age at diagnosis (years)					0.165
≤60	74.1	55.6	40.7	70.0	
>60	55.6	48.1	40.7	58.0	
AJCC stage					***0.015***
I/II	71.8	61.5	38.5	82.0	
III/IV	46.7	26.7	13.3	24.0	
T stage					0.238
T1/2	68.2	54.5	34.1	67.0	
T3/4	50.0	40.0	20.0	24.0	
Lymphatic metastasis					***0.003***
No	77.8	70.4	48.1	90.0	
Yes	51.9	33.3	14.8	36.0	
Tumor type					0.151
SC	62.5	56.3	28.1	62.0	
AC	71.4	47.6	38.1	58.0	
ASC	0	0	0	8.0	
MTA1 expression					***0.048***
Negative	84.6	76.9	53.8	Not reach	
Positive	58.5	43.9	24.4	42.0	

AJCC: American Joint Committee on Cancer.

SC: squamous carcinoma; AC adenocarcinoma; ASC: adeno-squamous carcinoma.

mOS, median overall survival time.

Significant *P*-values are bolded (*P*<0.05).

**Table 3:** Multivariate analysis of NSCLC patient prognosis (n=54)

Variable	P	HR	95.0% CI	
			Lower	Upper
AJCC C stage	0.267	1.521	0.725	3.188
Lymphatic metastasis	***0.021***	2.302	1.132	4.682
MTA1 expression	0.207	1.82	0.717	4.618

HR, hazard ratio; CI, confidence interval.

Significant *P*-values are bolded (*P*<0.05).

### MTA1 expression is positively correlated with that of p-AKT and β-catenin (cytoplasm), but not β-catenin (nucleus) in NSCLC tissues

We examined p-AKT and β-catenin expression in 56 NSCLC tissue samples. Correlations between MTA1 and p-AKT, as well as β-catenin are shown in Table 4 (Figure 8C). High MTA1 expression was positively associated with high p-AKT and β-catenin expression in the cytoplasm, but not β-catenin expression in the nucleus.

**Table 4:** Correlations between MTA1 expression and p-AKT or β-catenin expression in NSCLC tissues

MTA1	p-AKT				β-catenin (nucleus)				β-catenin (cytoplasm)			
	weak	high	*P*-value	V	weak	high	*P*-value	V	weak	high	*P*-value	V
Weak	10	5	***0.000***	0.545	14	1	0.082	0.055	11	4	***0.001***	0.406
High	5	36			28	13			6	35		

V, Cramer's V.

Significant *P*-values are bolded (*P*<0.05).

## DISCUSSION

MTA1 reportedly promotes cancer cell invasion and metastasis through E-cadherin expression regulation [17, 18, 27] and EMT [19–21]. However, the role of MTA1-induced EMT in NSCLC has not been thoroughly studied. Our results indicated that MTA1 upregulation promoted NSCLC cell migration and invasion, and inhibited cell adhesion. The opposite effects were observed in MTA1-silenced cancer cells, which was consistent with previous studies. Additionally, the present study confirmed that MTA1-induced NSCLC cell metastasis was related to EMT promotion *in vivo* and *in vitro*. We found that in NSCLC, MTA1 promoted EMT by activating AKT/GSK3β/β-catenin, but not Wnt/GSK3β/β-catenin signaling.

MK2206 treatment or AKT knockdown decreased MTA1 expression, indicating a positive feedback loop between MTA1 and p-AKT. The PI3K/AKT pathway is constitutively activated in NSCLC cells [26]. NSCLC cells treated with MK2206 exhibited increased adhesion and decreased migration and invasion. suggesting that targeting AKT or both MTA1 and AKT may be a promising anti-NSCLC therapeutic strategy.

However, MK2206 appeared to have no effect on invasion or migration in the normal lung cell line, Beas-2b, which may due to the endogenous AKT activity. The pAKT expression was negative or weak in normal lung tissues.

We found that high MTA1 expression in NSCLC patient tissues was positively correlated with high cytoplasmic p-AKT and β-catenin expression. This suggested that MTA1 might activate AKT and therefore AKT/GSK3β/β-catenin signaling, thereby promoting metastasis. Our results supported a new role for MTA1 in promoting EMT, a key metastasis-related process [2–4]. An understanding of the MTA1-AKT interaction molecular mechanism will require further study. MTA1 was recently reported to regulate PTEN acetylation and, indirectly, AKT activation [35]. The present study found that MK2206 did not completely reverse effects associated with MTA1 expression changes, indicating that pathways besides AKT/GSK3β/β-catenin signaling could be involved in MTA1-induceed EMT in NSCLC.

In summary, our results indicated that MTA1 promotes NSCLC cell EMT by activating AKT/GSK3β/β-catenin signaling, indicating that MTA1 is a potential anti-NSCLC therapeutic target. Due to the positive feedback loop between MTA1 and p-AKT, blocking both MTA1 and p-AKT may represent a novel therapeutic strategy for cancer treatment.

## MATERIALS AND METHODS

### Plasmids, shRNA, siRNA, and reagents

The plasmid, pCMV-MTA1-EGFP-SV40-Neomycin, and the negative control empty plasmid, pCMV-EGFP-SV40-Neomycin, were purchased from GeneChem Co., Ltd. (Shanghai, China). For MTA1 overexpression, the full-length MTA1 cDNA was obtained by PCR amplification using the following primers: forward, 5′-TACCGGACTCAGATCTCGAGATGGCCGCCAACATGTACAG-3′; reverse, 5′-GATCCCGGGCCCGCGGTACCGTGTCCTCGATGACGATGGGCTC-3′. The PCR product was cloned into the XhoI and Kpnl restriction sites of the expression vector, GV230, to produce the plasmid, pCMV-MTA1-EGFP-SV40-Neomycin.

Lentivirus-mediated shRNAs targeting MTA1 (LV-shRNA), small interfering RNAs (siRNA) targeting AKT and the corresponding negative controls were also designed and synthesized by GenePharma Co., Ltd. (Shanghai, China). To knock down endogenous MTA1, the following target sequences were cloned: sh#1(1198): 5′AATTCAAAAAAGCAGCAGAAACGCTTGAAAGC TCTCTTGAAGCTTTCAAGCGTTTCTGCTGCG-3′; sh#2(1437): 5′AATTCAAAAAAGCGCATCTTGTTGGACATATTCTCTTGAAATATGTCCAACAAGATGCGCG-3′; sh#3(680): 5′AATTCAAAAAAGGAGAGATTCGAGTAGGAAACTCTCTTGAAGTTTCCTACTCGAATCTCTCCG-3′; control sh: 5′-TTCTCCGAACGTGTCACGT-3′. To knock down endogenous AKT, the following target sequences were constructed in a small interfering RNA (siRNA) vector: siRNA#1- AKT: sense: 5′-GCUAUUGUGAAGGAGGGUUTT-3′, antisense: 5′-AACCCUCCUUCACAAUAGCTT-3′; siRNA#2- AKT: sense: 5′- GGCCCAACACCUUCAUCAUTT-3′, antisense: 5′- AUGAUGAAGGUGUUGGGCCTT-3′. A scrambled siRNA sequence was used as a negative control: 5′-UUCUCCGAACGUGUCACGUTT-3′, antisense: 5′- ACGUGACACGUUCGGAGAATT-3′. The AKT inhibitor, MK-2206 2HCl (S1078), was purchased from Selleckchem (Houston, TX, USA).

### NSCLC tissue samples

Clinical and pathological data were retrospectively collected from 56 patients diagnosed with NSCLC at the First Affiliated Hospital of Xi'an Jiaotong University between Jan 2005 and Dec 2007. Patients included 46 males and 10 females aged 32–79 years (mean age, 59.16 years). No patient received anti-cancer treatment prior to tumor excision. All patients were classified according to the p-TNM staging system of the American Joint Committee on Cancer stage [36] and the classification system of the World Health Organization [37]. Patients were followed up until death or the end of the study (December 2015). Survival time was calculated from the date of diagnosis to death or the end of follow-up. The clinical study was approved by the Ethics Committees of the First Affiliated Hospital of Xi'an Jiaotong University. For immunohistochemistry, 20 normal lung tissues were used as controls.

### Cell culture

Human NSCLC cell lines, H460, A549, and 95-D, as well as the normal human lung epithelial cell line, Beas-2b, were kindly provided by the Translational Medical Center of the Medical College of Xi'an Jiaotong University. Beas-2b cells were cultured in DMEM (HyClone, Logan, UT, USA). NSCLC cells were cultured in RPMI-1640 medium (HyClone). Cell culture media contained 10% fetal bovine serum (FBS; HyClone), 100 U/ml penicillin and 100 μg/mL streptomycin (Life Technologies, Grand Island, NY, USA). Cells were cultured at 37°C with 5% CO_2_.

### RT-PCR

Total RNA was extracted from cells using the Fast 200 RNA Extraction Kit (Feijie Biological Technology, Shanghai, China), and 1 μg of total RNA was used to synthesize cDNA using a Reverse Transcription Kit (#RR036A; Takara, Dalian, China) according to the manufacturer's instructions. RT-PCR primer sequences were as follows: MTA1: forward: 5′-GACCAGGCAGGCTTTCTATC-3′, reverse: 5′-CTG TTGATGGGCAGGTAGG-3′; GAPDH: forward: 5′-AGG TCCACCACTGACACGTT-3′, reverse: 5′-GCCTCAA GATCAGCAAT-3′. PCR products were separated electrophoretically on 2% agarose gels, and bands were visualized using ethidium bromide staining. Band intensity was evaluated using an agarose gel imaging system (Bio-Rad, Hercules, CA, USA).

### Western blotting

Total protein was extracted using cell lysis buffer containing protease and phosphatase inhibitors (Roche, Indianapolis, IN, USA). Samples with equal total protein amounts were separated by sodium dodecyl sulfate-polyacrylamide gel electrophoresis (SDS-PAGE) and transferred onto PVDF membranes (Millipore Corp., Boston, MA). The following primary antibodies were used: MTA1 (Cell Signaling, #5647, 1:1,000), E-cadherin (Proteintech, 20874-1-AP, 1:1,000), Vimentin (Proteintech, 10366-1-AP, 1:2,000), Claudin-1 (Cell Signaling, #13255, 1:1,000), ZO-1 (Cell Signaling, #8193, 1:1,000), p-AKT (WLP001a, 1:750), AKT (WL0003b, 1:750), p-GSK3β (Cell Signaling, #9323,1:1,000), GSK3β (Cell Signaling #9315, 1:1,000), β-catenin (Proteintech, 51067-2-AP, 1:1,000), GAPDH (HRP-60004, 1:5,000), and Wnt1 (Sangon Biotech, D261302, 1:50) Secondary antibody (Cell Signaling, #7044) was diluted 1:2,000. Signals were visualized using the ECL detection system (Millipore Corp., Boston, MA) according to the manufacturer's instructions.

### Confocal microscopy

For immunofluorescence staining, cells were seeded into 24-well plates with glass coverslips and fixed with 4% paraformaldehyde in PBS. Cell membranes were permeabilized with 0.5% Triton X-100 in PBS, and nonspecific binding sites were blocked with 5% bovine serum albumin (BSA) in PBS. Slides were incubated with the first antibody, MTA1 (Abcam, ab71153; 1:200), at 4°C overnight. After three washes with cold PBS, cells were incubated with Cy3-conjugated IgG (EK022, 1:200 dilution in PBS) at room temperature for 30 min. After three washes with cold PBS, cells were sealed with a fluorescence quenching sealing tablet containing DAPI (36308ES11, Yeasen) and examined under a confocal microscope (Olympus, Tokyo, Japan)., TRITC Phalloidin (100 nM; 40734ES75, Yeasen, Shanghai, China) was used for cell structure staining.

### Plasmid transfection

Beas-2b and H460 cells were transfected with overexpression plasmid, pCMV-MTA1-EGFP-SV40-Neomycin, and control empty plasmid, pCMV-EGFP-SV40-Neomycin, using TurboFect Transfection Reagent (#R0531, Thermo Scientific, Waltham, MA, USA) at 1:2 (plasmid:Transfection Reagent, μg/μL) according to the manufacturer's instructions. Transfected cells were maintained in appropriate growth medium supplemented with 800 μg/mL G418 for 14 d to select neomycin-resistant colonies. For transient infection, cells were transfected for 72 h without G418 selection.

### Lentivirus-mediated shRNA infection

95-D and A549 cells were infected by lentiviral particles at a multiplicity of infection (MOI) of 5 with 5 μg/ml polybrene, followed by puromycin selection (95-D at 10 μg/mL, A549 at 2.0 μg/mL) for 10 d to select stably-expressing cells. For transient infection, cells were infected for 72 h without puromycin selection.

### Small interfering RNA (siRNA) transfection

H460 cells were transfected with siRNA or plasmid using the X-tremeGENE siRNA Transfection Reagent (Roche, Indianapolis, IN, USA) at 5:1 (X-tremeGENE siRNA Transfection Reagent:siRNA/plasmid; μg:μg). For co-transfection, cells were transfected with MTA1-overexpression plasmid for 24 h, then transfected with siRNA for another 48 h. Cells were collected after 72 h total.

### Cell adhesion assay

Cells were seeded into 96-well plates precoated with 0.04 μg/μL Matrigel (BD, San Diego, USA) for 2 h. Plates were immersed in PBS to remove non-adherent cells. 20 μL MTT [3-(4, 5-dimethylthiazol-2-yl)-2, 5-diphenyl tetrazolium bromide] (5 mg/mL, Promega, Shanghai, China) was added to each well and incubated at 37°C in darkness for 4 h. After supernatant removal, 100 μL dimethyl sulfoxide (DMSO; Sigma-Aldrich, St. Louis, MO, USA) was added to each well to dissolve the formazan product. Absorbance at 490 nm was recorded with a Microplate Reader (Bio-Rad, Hercules, CA, USA). The adhesion rate was calculated using the following equation: OD of the experiment/OD of the control×100%.

### Wound healing assay

The cell monolayer was scratched using a sterile 10-μL pipette tip and washed with PBS to remove detached cells. The remaining cells were cultured in serum-free medium, and photos were taken at 0 and 36 h. Gap widths were measured using IPP 6.0 software (Media Cybernetics, Bethesda, MD, USA), and data acquired from three areas of the wound on each plate were used to calculate the mean gap width at a given time.

### Transwell invasion and migration assay

Cell migration and invasion were performed using Transwell plates (8-μm pore size, Corning) without Matrigel (for migration assays) or with Matrigel (for invasion assays). Briefly, prepared cells (invasion 5×10^4^; migration 2×10^4^) were plated onto upper chambers with serum-free medium. RPMI-1640/ DMEM medium containing 10% FBS was added to the bottom chamber. After 36 h incubation, non-invading cells in the upper chamber were removed and invasive cells in the lower chamber were fixed with 4% paraformaldehyde and stained with crystal violet. The number of invasive cells was quantified by counting the number of cells in five randomly chosen fields at 200x magnification.

### Animal model

Four-week-old male athymic BALB/c nude mice were purchased from the Animal Center of the Medical College of Xi'an Jiaotong University, Xi'an, China and housed under specific pathogen-free conditions. Animals were randomly assigned to four groups (5 animals/group), which were administered equal numbers (1×10^6^) of MTA1oex/H460, Control/H460, MTA1-sh/A549, Control-sh/A549, MTA1-sh/95D, or Control-sh/95D cells via tail vein injection. 25 d following injection, mice were euthanized. Lung tissues were removed, fixed in 10% formalin, and embedded in paraffin for pathological analysis. One mouse in the MTA1oex/H460 group died on d 24. All animal experimental procedures were carried out according to protocols approved by the Ethics Committee for Animal Experimentation of the Medical College of Xi'an Jiaotong University, in accordance with the National Institutes of Health Guide for the Care and Use of Laboratory Animals.

### Immunohistochemistry

Fixed xenograft lung tissues and NSCLC lung tissue specimens were cut into 4-μm serial sections. After antigen retrieval (using a microwave at high power for eight min, followed by middle-low power in 10 mM of citrate buffer, pH 6.0 for 13 min), sections were treated to quench endogenous peroxidase activity. Immunohistochemistry staining was then performed using a Streptavidin–biotin peroxidase (SP) kit (SP-9001; Beijing Zhongshan Golden Bridge Biotechnology, China) according to the manufacturer's instructions. Sections were immunostained with primary antibodies as follows: MTA1 (ab71153, 1:200), E-cadherin (20874-1-AP, 1:50), Vimentin (10366-1-AP, 1:200), Claudin-1 (WL00448, 1:50), and ZO-1 (21773-1-AP, 1:100). Expression intensities were quantified as the sum of the integrated optical densities (IOD)/sum of the area of threshold pixels for all signals measured in each image. Imaging analysis was performed using IPP 6.0 software (Media Cybernetics, Bethesda, MD, USA). Results were expressed as means ± standard error (means ± SEM).

Sections from 56 NSCLC patient lung tissues and 20 normal lung tissues were incubated with the following primary antibodies: MTA1 (ab71153, 1:100), p-AKT (WLP001a, 1:100), and β-catenin (51067-2-AP, 1:100). The proportion of stained cells in each field was assessed as follows: 0, no stained cells; 1, 1–10% stained cells; 2, 11–20% stained cells; 3, 21–50% stained cells; and 4, >50% stained cells. Staining intensity was graded as follows: 0, negative staining; 1, light staining; 2, moderate staining; and 3, intense staining. The staining intensity distribution (SID; the score of the proportion of the stained cells was multiplied by the score of the staining intensity) was judged as follows: -, score 0; +, score 1–3; ++, score 4–6; and +++, score 7–12. Patients who scored 0–3 points were identified as having weak expression; those with 4–12 points had high expression.

### Statistical analysis

Statistical analysis was performed using SPSS software (Statistical Package for the Social Sciences version 21.0; SPPS Inc., Chicago, IL, USA). The data were expressed as the means ± SEM. Differences between two groups were analyzed using the student t-test. Differences between three or more groups were analyzed using one-way ANOVA and least-significant difference (LSD). Chi-square test was used to analyze differences between clinicalpathological variables, Kaplan-Meier estimates and log-rank tests were used for survival analysis, and Cox proportional hazards regression model was used to identify independent factors associated with prognosis. All statistical tests were two sided. *P*<0.05 was considered statistically significant.
